# Salinity-Linked Denitrification Potential in Endorheic Lake Bosten (China) and Its Sensitivity to Climate Change

**DOI:** 10.3389/fmicb.2022.922546

**Published:** 2022-07-14

**Authors:** Xingyu Jiang, Changqing Liu, Yang Hu, Keqiang Shao, Xiangming Tang, Guang Gao, Boqiang Qin

**Affiliations:** ^1^State Key Laboratory of Lake Science and Environment, Nanjing Institute of Geography and Limnology, Chinese Academy of Science, Nanjing, China; ^2^University of Chinese Academy of Sciences, Beijing, China

**Keywords:** nitrate availability, salinity, arid region, Northwest China, species diversity, endorheic lake

## Abstract

Endorheic lakes in arid regions of Northwest China are generally vulnerable and sensitive to accelerated climate change and extensive human activities. Therefore, a better understanding of the self-purification capacity of ecosystems, such as denitrification, is necessary to effectively protect these water resources. In the present study, we measured unamended and amended denitrification rates of Lake Bosten by adding the ambient and extra nitrate isotopes in slurry incubations. Meanwhile, we investigated the abundances and community structure of nitrous oxide-reducing microorganisms using qPCR and high-throughput sequencing, respectively, in the surface sediments of Lake Bosten to study denitrification potential in endorheic lakes of arid regions as well as the response of those denitrifiers to climatically induced changes in lake environments. Amended denitrification rates increased by one order of magnitude compared to unamended rates in Lake Bosten. The great discrepancy between unamended and amended rates was attributed to low nitrate availability, indicating that Lake Bosten is not operating at maximum capacity of denitrification. Salinity shaped the spatial heterogeneity of denitrification potential through changes in the abundances and species diversity of denitrifiers. Climate change had a positive effect on the water quality of Lake Bosten so far, through increased runoff, decreased salinity, and enhanced denitrification. But the long-term trajectories of water quality are difficult to predict alongside future glacier shrinkage and decreased snow cover.

## Introduction

Endorheic lakes, as one of the major components of endorheic water systems, are the primary available water sources in arid regions and play significant roles in the social and economic development of those regions (Tao et al., 2015; Xu et al., 2020). High degrees of continentality and isolation make endorheic lakes particularly susceptible and vulnerable to climate and environmental change (Yapiyev et al., 2017; Huang et al., 2020). Due to the discharge of agricultural, industrial, and urban wastewaters, endorheic waters have been increasingly threatened by eutrophication in recent decades (Arce et al., 2013; Menberu et al., 2021; Sun et al., 2021; Diaz-Torres et al., 2022). More importantly, endorheic lakes are land-locked drainage networks where water does not drain into other water bodies (Yapiyev et al., 2017). Due to the lack of outlets, the removal of inflowing pollutants depend primarily on the self-purification capacity of ecosystems (Arce et al., 2013; Valiente et al., 2018). Therefore, a better understanding of the self-purification capacity and ecological resilience of endorheic lakes is necessary to effectively protect the water resources in arid regions.

Denitrification, the process of reducing nitrate to dinitrogen (N_2_), is considered the primary pathway for permanent nitrogen (N) removal in aquatic ecosystems (Seitzinger, 1988). It plays an important role in alleviating N pollution and maintaining the self-purification capacity of ecosystems (Roland et al., 2018). The availability of both nitrate and organic carbon are considered the most important factors limiting denitrification rates (Seitzinger, 1988; Seitzinger et al., 2006; Xia et al., 2017; Jiang et al., 2020). Salinity also has an important influence on denitrification (Koop-Jakobsen and Giblin, 2010; Arce et al., 2013; Zhou et al., 2016). Many endorheic lakes are naturally saline, as the evaporative concentration process leads to salt accumulation (Yapiyev et al., 2017), which comprises a diverse array of salts such as calcium, sodium, potassium, sulfate, carbonate, and chloride (Heinrichs and Walker, 2006). The change in salinity can cause decrease or increase in cytoplasmic volume by imposing considerable osmotic stress on the relevant microbes such as nitrifiers and denitrifiers, resulting in the loss of metabolic activity (Ardon et al., 2013; Zhao et al., 2013b; Neubauer et al., 2019). In addition, salt water containing sulfate may have inhibitory or promoting effects on nitrate reduction due to sulfide toxicity or by providing electron donors for chemoautotrophic processes from the reduction of sulfate (Aelion and Warttinger, 2010; Zhu et al., 2018; Murphy et al., 2020). A consensus on these mechanisms remains elusive, probably due to the differences in microbial community structure involved in N cycling, which is sensitive to salinity variation (Herbert et al., 2015; Zhou et al., 2016). Lakes are hotspots of denitrification with high N removal efficiency due to the long residence times of water (Wollheim et al., 2008; Finlay et al., 2013). Previous research about denitrification has mainly focused on out-flowing lakes. We lack a direct understanding of the denitrification potential of endorheic lakes in arid regions.

Climate change introduces a new challenge for endorheic lakes (Tao et al., 2015; Zhang et al., 2017). In Northwest China, temperatures have risen markedly in recent decades and faster than in the surrounding regions (Shi et al., 2007; Yang et al., 2020). Climate change can influence lakes either through higher temperatures or by changes in salinity (Mosley, 2015; Greaver et al., 2016), the latter is particularly influential in arid and semiarid lakes (Brucet et al., 2012; Lin et al., 2017). Climate change is affecting the hydrological cycle with more frequent and intense precipitation, altered snow accumulation and melt, and changes in evaporation (Sorg et al., 2012; Zhou et al., 2015), leading to large-scale changes in lake salinity (Jeppesen et al., 2015; Rusuli et al., 2015). Thus, how denitrification in endorheic lakes will respond to climatically induced changes in lake salinity is a key question.

Lake Bosten used to be the largest freshwater lake in the endorheic basins of China and is of great importance as a water supply for local domestic use and industrial and agricultural production (Zhou et al., 2015; Wang et al., 2018b). Since the 1960s, Lake Bosten suffers from salinization and eutrophication mainly caused by accelerated climate change and extensive human activities (Guo et al., 2015b; Fontana et al., 2019). It has changed from an oligotrophic freshwater lake to a mesotrophic oligosaline lake (Tang et al., 2012; Dai et al., 2013). Lake Bosten represents an interesting feature, which has a natural salinity gradient (Tang et al., 2012; Dai et al., 2013). It provides an ideal ecosystem in which to study the denitrification capacity of endorheic lakes in arid regions as well as its response to climatically induced changes in lake salinity.

Slurry incubations incorporating the ^15^N isotope-tracing technique have been used as a powerful tool for estimating potential denitrification rates in the field (Song et al., 2013; Yin et al., 2014; Jiang et al., 2020) and simulation experiments (Salk et al., 2017; Wang et al., 2018a; Murphy et al., 2020). Although denitrification rates in slurries are usually significantly higher than under *in situ* conditions due to the disruption of natural profiles of sediments (Laverman et al., 2006), slurry incubation is highly repeatable and flexible (Yin et al., 2014; Salk et al., 2017). It is suitable for exploring the relative differences in denitrification potential and estimating nitrate limitations by adding extra nitrate (amended) and not adding extra nitrate (unamended) to the slurries. During denitrification, nitrite and nitrous oxide reductase enzymes can catalyze the reduction of nitrite and nitrous oxide, which are encoded by the *nirS*/*nirK* and *nosZI*/*nosZII* genes, respectively (Franklin et al., 2017; Neubauer et al., 2019; Murphy et al., 2020). These genes commonly used as an amplicon for quantifying or sequencing denitrifiers (Throback et al., 2004). Quantifying the potential denitrification rates, combined with the abundance of key genes, can provide a comprehensive understanding of lake denitrification potential (Murphy et al., 2020; Broman et al., 2021; Yang et al., 2022).

Here, we examine unamended and amended denitrification rates at 17 sampling sites in Lake Bosten through the addition of extra nitrate (100 μmol L^−1^) or otherwise to slurries, in order to estimate potential denitrification rates. We determine the abundance of *nirS* and *nosZI* genes in surface sediments through qPCR to estimate the genetic potential of denitrification. In addition, high throughput sequencing using *nosZI* gene as an amplicon was conducted to estimate the microbial community structure mediating denitrification. We test the following hypotheses: (1) low nitrate concentration may be a proximate control of denitrification rates in Lake Bosten; (2) the sediment of different areas in Lake Bosten varies in denitrification potential due to the difference in nitrate and salinity levels; and (3) based on earlier studies about the influence of salinity on denitrification, we hypothesize that climate change can influence lake denitrification *via* changes in salinity. Our study provides insights into the influence of changes in salinity on N cycling in endorheic lakes and can be used to predict future response of water quality to climate change in arid regions.

## Materials and Methods

### Study Area

Lake Bosten is located in the lowest area of the intermontane Yanqi Basin between the Taklimakan Desert and Tien Shan, Northwestern China. Winds come mainly from the southwest, indicating dominant influences of westerlies throughout the summer season (Ma et al., 2020; Yao et al., 2022). The mean annual temperature was ~9.1°C, and the annual precipitation was 76.3 mm. Lake Bosten has a surface area of ~1,005 km^2^, a maximum depth of 16 m, and an average depth of 8 m. Water temperature and pH ranged from 23.5 to 24.9°C and from 8.8 to 9.5, respectively (Supplementary Table S1).

The Kaidu River is the most important tributary in the area and accounts for about 83% of the lake's annual water inflow runoff, which is supplied by melting ice, precipitation, and groundwater (Chen et al., 2008; Rusuli et al., 2016). Other rivers are small seasonal rivers, such as the Huangshui River, which contributes to almost all the remaining 17% of inflow runoff (Chen et al., 2008; Rusuli et al., 2016). A pumping station was constructed in the southwestern part of Lake Bosten, which pumps lake water into the Peacock River to adjust the outflow of the lake (Yu et al., 2015; Zhou et al., 2017).

**Figure 1:**
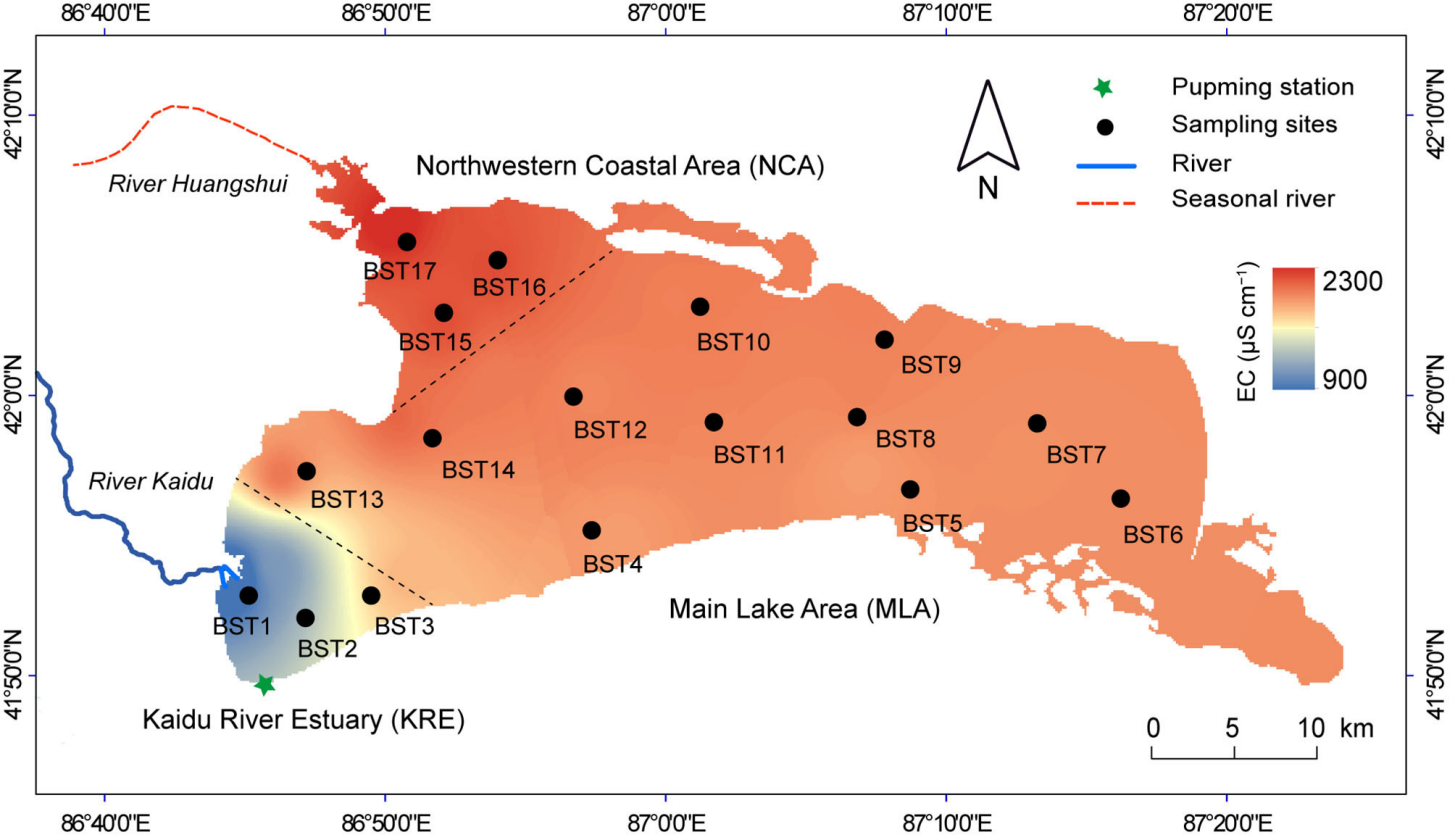
Map of Lake Bosten, showing study area, sampling sites and electrical conductivity (EC) in water column. Electrical conductivity was the average value from 2010 to 2019, which derived from the long-term monitoring of the institute of Lake Bosten. The lake area was divided into three subregions according to the EC gradient: Kaidu River Estuary (KRE), Main Lake Area (MLA) and Northwestern Coastal Area (NCA).

The freshwater inflow from the Kaidu River is removed by the pumping station at the southwestern margin of Lake Bosten, shaping a freshwater region in the southwest of Lake Bosten (Kaidu River Estuary: KRE). The drainage channels of agricultural production are mainly located in the northwestern coastal area (NCA), making this area the most heavily polluted in Lake Bosten. The rest of the area is the main lake area (MLA) (Figure 1).

### Collection and Pre-Treatment of Samples

Water and sediment samples, from 17 sites at Lake Bosten, were collected in September 2019. Water samples from the top (50 cm below the surface), middle, and bottom (50 cm above the sediment) were collected using a polymethyl methacrylate water sampler (UWITEC, Austria) and then mixed. Water samples for dissolved nutrient analyses were filtered using 0.2 μm nylon syringe filters immediately following the mixture in the field. The surface sediments (about 3 cm depth) were collected with a 60 cm long gravity corer (UWITEC, Austria), and then placed in clean airtight plastic bags. Water temperature (WT), pH, dissolved oxygen (DO), and salinity were measured at each sampling site *in situ*, with a multiparameter water quality sonde (YSI 6600V2, USA). All collected sediment and water samples were kept in a cool and shaded place, and subsequently delivered to the Institute of Lake Bosten.

### Measurements of Unamended and Amended Denitrification Rates

Unamended and amended denitrification rates were measured using slurry incubations incorporating the ^15^N isotope-tracing technique (Yin et al., 2014; Jiang et al., 2020). Sediment samples were mixed with lake water at a ratio of 1:10 to make homogenized slurries. The slurry was stirred continuously and purged with helium for 40 min. The purged slurries were distributed into 12 ml vials (Labco Exetainer, UK), and then immediately sealed. Subsequently, preincubation was conducted at the *in situ* temperature on a shaker table (200 rpm) for 24 h, to eliminate residual nitrate and DO. After preincubation, a certain amount of ^15^N-nitrate (99 atom%) solution was injected into a group of vials to determine the unamended denitrification rates according to the nitrate concentrations of the water column in Lake Bosten. Simultaneously, a high ^15^N-nitrate level (a final concentration of 100 μmol L^−1^) was injected into another group of vials to estimate the amended potential rates. Subsequently, three of the vials were randomly selected from each group and immediately preserved with the addition of 200 μL ZnCl_2_ as the initial samples for the measurement of denitrification rates. The remaining vials were incubated under the same conditions for 2, 4, and 8 h, and then used to measure the production of ^29^N_2_ and ^30^N_2_.

Dissolved ^29^N_2_ and ^30^N_2_ were determined using membrane inlet mass spectrometry (MIMS) analysis (Kana et al., 1998). The unamended and amended denitrification rates were calculated according to the following equation:


(1) $$R=\frac{K\times V}{W}$$

where *R* (μmol N kg^−1^ h^−1^) indicates the measured ^15^N-based unamended or amended denitrification rates, *K* is the slope calculated from the concentration of ^15^N-N_2_ vs. incubation time (Supplementary Figure S1), *V* (L) is the volume of the incubation vial, and *W* (kg) denotes the dry weight of the sediment.

### DNA Extraction and Real-Time Quantitative PCR (qPCR)

Genomic DNA was extracted from the sediment samples using the FastDNA™ Spin kit for soil (MP Biomedicals) according to the manufacturer's instructions. The quality and quantity of DNA were checked using agarose gel electrophoresis and using a NanoDrop ND-1000 UV/Vis spectral photometer. The quality of DNA extracted in each sampling site was exhibited in Supplementary Table S2. Extracts were stored at −80°C prior to gene quantification. The sampling site of BST06 and BST13 were removed from the following molecular biological analysis due to low DNA quality.

qPCR was used to quantify functional genes for enzymes of nitrification (*AOB* and *AOA*) (Rotthauwe et al., 1997; Francis et al., 2005) and denitrification (*nirS* and *nosZI*) (Throback et al., 2004). Triplicate qPCR reactions were set up for each sample. The primers, thermocycling conditions, and relevant references are included in the supplemental information (Supplementary Table S3). The total qPCR reaction volume of 20 μl contained 10 μl of SYBR green qPCR Master Mix, 1 μl of forward primer, 1 μl of reverse primer, 7 μl of ddH_2_O, and 1 μl of the template (DNA). Reactions were performed using an Eco™ Real-Time PCR System. Melting curves were checked for each reaction to confirm the purity of the amplified products. Standard curves were obtained using 10-fold serial gradient dilutions of standard plasmids containing targeted genes with known copy numbers. The qPCR amplification efficiencies and other calibration curve parameters are listed in Supplementary Table S4. Gene abundance was calculated based on the constructed standard curve and then converted into copies per gram of dry sediment.

### High-throughput Sequencing

The microbial reduction of nitrous oxide to N_2_ is catalyzed by nitrous oxide reductase. The *nosZI* gene was sequenced to explore the microbial community structure performing complete denitrification across the lake using the primer pairs *nosZ-F*/*nosZ1622R* (Throback et al., 2004). The purified DNA products were sent to Shanghai Personal Biotechnology Co., Ltd for high-throughput sequencing using the Illumina MiSeq platform. Paired-end sequencing reads were merged using FLASH (Fast Length Adjustment of Short reads, v1.2.11) (Magoc and Salzberg, 2011). Adapters and primers were trimmed off all the reads using Cutadapt (v1.9.1). Sequences shorter than 400 bp, lower than 25 quality scores, and suspected to be chimeras were discarded using USEARCH (v10.0.240) (Edgar and Flyvbjerg, 2015). The sequences were initially grouped at 97% of the sequence similarities, and representative sequences were translated and compared to the *nosZ* reference sequence in GenBank using BLASTx. Frameshift errors were removed. Subsequently, the high-quality sequences were aligned based on the amino acid residues and grouped based on 85% of the nucleotide sequence similarities to form a new OTU table for the subsequent analysis.

### Chemical Analysis of Water and Sediment Physicochemical Parameters

Dissolved organic carbon (DOC) was determined using a TOC analyzer (Teledyne Tekmar, Torch, USA). Acid volatile sulfides (AVS) in sediments were determined with the following methods. Sulfide in sediment samples was converted into H_2_S by HCl extraction for 1 h. Subsequently, released H_2_S was captured in a NaOH solution with a continuous N_2_ flow. The dissolved sulfide concentration in the NaOH solution was measured spectrophotometrically by the methylene blue method. The other physicochemical parameters, including total nitrogen (TN), nitrate, ammonium, sulfate, sediment total organic carbon (STOC), and sediment total nitrogen (STN) were determined following standard methods detailed in Jiang et al. (2020).

### Statistical Analyses

The statistical analyses were performed using R 3.5.3 and the RStudio 1.1.462 interface. Kruskal–Wallis rank tests and one-way ANOVA were used to evaluate the differences in environmental factors, gene abundances, species diversity, and denitrification rates among areas, determining statistically significant differences when *p* < 0.05. Alpha diversity indices, unconstrained PCoA analysis, and redundancy analysis (RDA) were constructed with the “vegan” package in R. Pearson correlations were used to examine the relationships between parameters of denitrification and environmental properties. Data visualization was performed using the package “ggplot2” in R.

## Results

### The Physicochemical Parameters of the Water and Sediment in Lake Bosten

**Figure 2:**
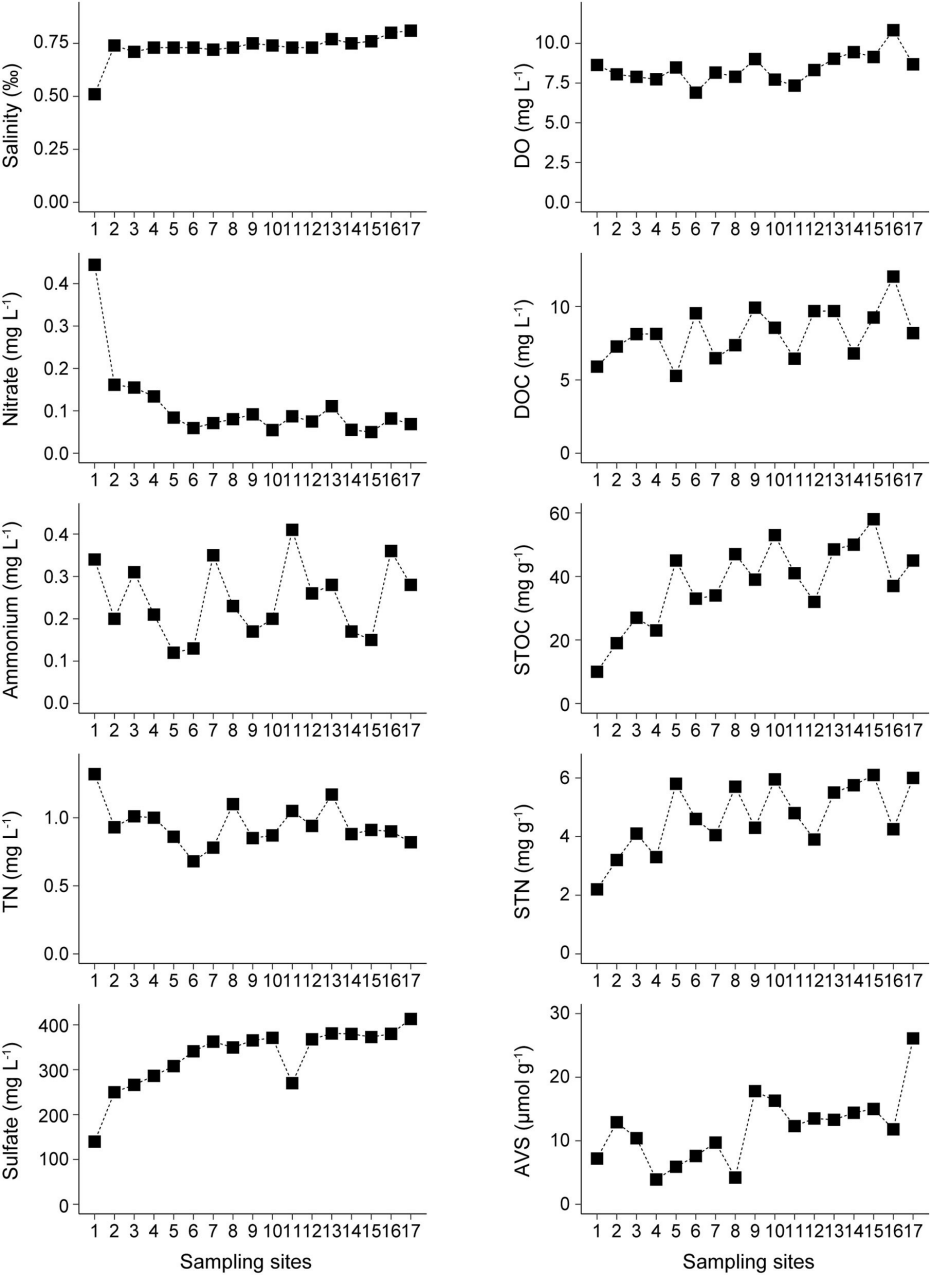
Spatial distribution of the physicochemical parameters in the overlying water [Salinity, Dissolved Oxygen (DO), Nitrate, Ammonium, Total Nitrogen (TN), Sulfate and Dissolved Organic Carbon (DOC)] and sediments [Sediment Total Organic Carbon (STOC), Sediment Total Nitrogen (STN) and Acid volatile sulfides (AVS)] in Lake Bosten.

In Lake Bosten, dissolved oxygen and salinity ranged from 6.9 to 10.8 mg L^−1^ and from 0.51‰ to 0.81‰, respectively. Dissolved oxygen in NCA was significantly higher than that in KRE and MLA, while salinity in KRE was significantly lower than that in MLA and NCA. The concentrations of nitrate and TN exhibited a decreasing trend from KRE to NCA. Especially for nitrate, the values in KRE were 3–4 times higher than the values in MLA and NCA, which ranged from 0.1 to 0.4 mg L^−1^. The concentrations of TN ranged from 0.7 to 1.3 mg L^−1^, and the values in KRE were significantly higher than that in NCA. In contrast, the opposite trend was found for sulfate, DOC, STOC, STN, and AVS, which ranged from 140 to 413 mg L^−1^, from 5.3 to 12.0 mg L^−1^, from 10.0 to 58.3 mg g^−1^, from 2.2 to 6.1 mg g^−1^ and from 3.9 to 26.1 μmol g^−1^, respectively. Ammonium concentrations ranged from 0.1 to 0.4 mg L^−1^, and there was no significant variation among the three areas in Lake Bosten (Figure 2).

### Unamended and Amended Denitrification Rates

**Figure 3:**
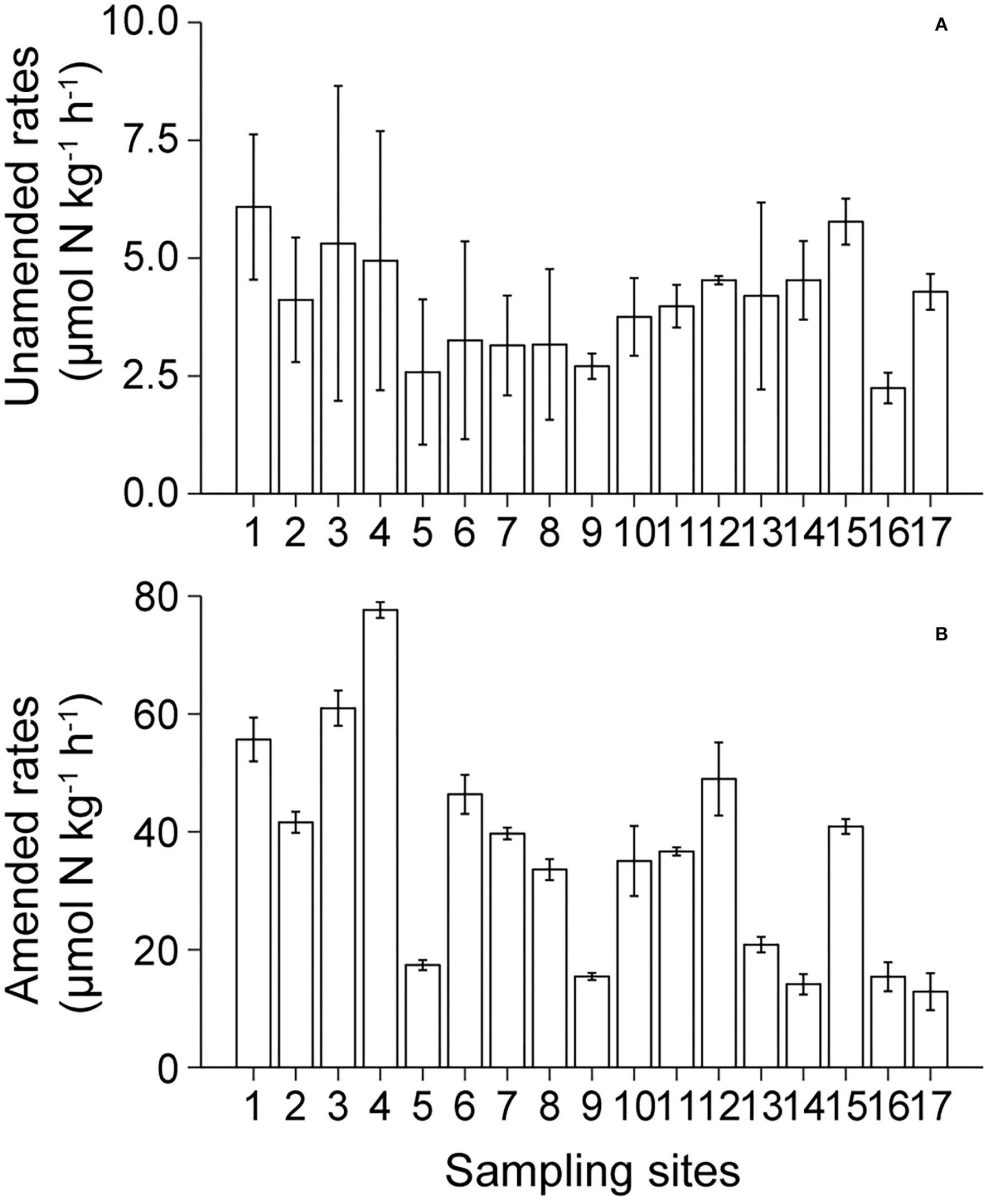
Spatial distribution of the unamended **(A)** and amended **(B)** denitrification rates in Lake Bosten.

Unamended and amended denitrification rates ranged from 2.24 to 6.08 μmol N kg^−1^ h^−1^ and from 12.87 to 77.63 μmol N kg^−1^ h^−1^, respectively, in Lake Bosten, and the amended rates were about 8.9 times those of the unamended rates (Figures 3A,B). These sampling sites showed considerable variation in amended denitrification rates. Generally, the amended rates were highest in KRE, intermediate in MLA, and lowest in NCA. In contrast, in unamended denitrification rates, the differences were small among sampling sites in Lake Bosten.

### The Abundance of Functional Genes for Nitrification and Denitrification

**Figure 4:**
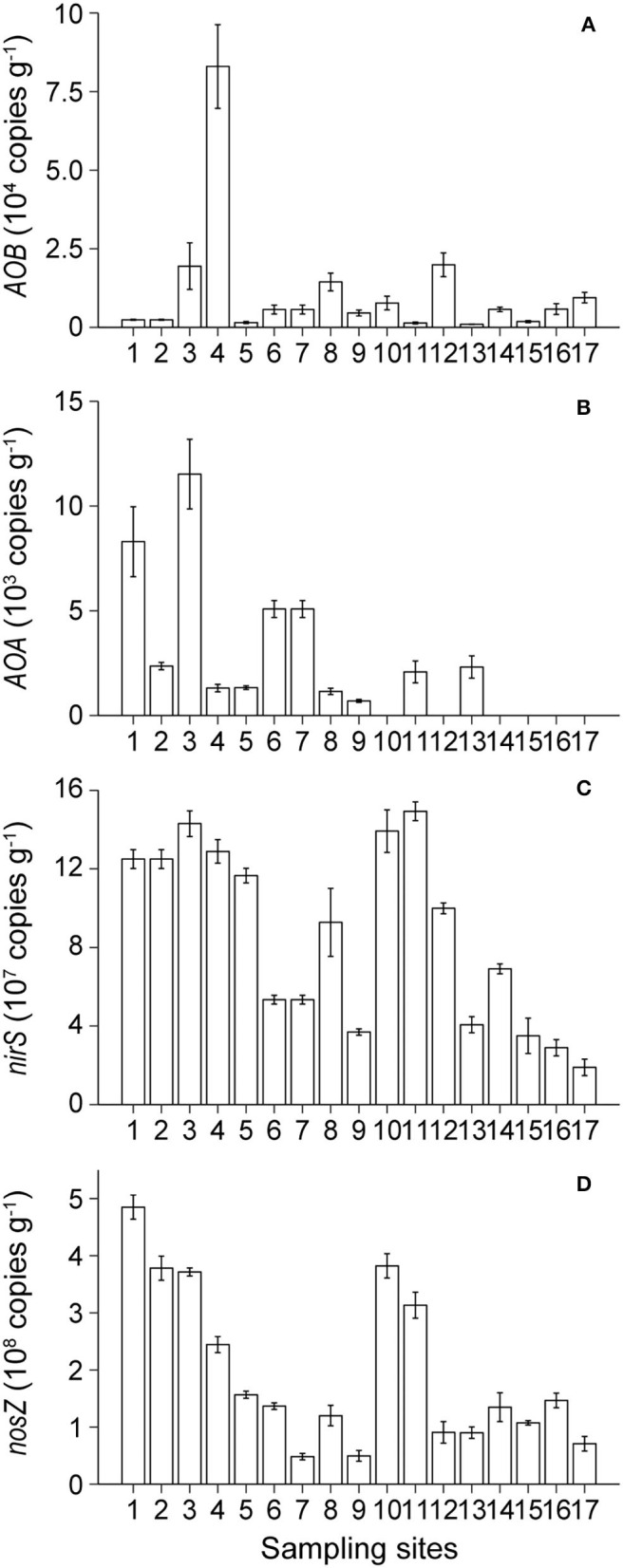
Spatial distribution of the abundances of *AOB* **(A)**, *AOA* **(B)**, *nirS* **(C)**, and *nosZI* **(D)** genes in Lake Bosten.

The bacterial amoA gene copy numbers ranged from 9.45 × 10^2^ copies g^−1^ of sediment at site BST13 to 8.30 × 10^4^ copies g^−1^ of sediment at site BST4 (Figure 4A). The archaeal amoA gene copy numbers ranged from undetected values to 1.15 × 10^4^ copies g^−1^ of sediment, and there was a decreasing trend from KRE to NCA (Figure 4B). The abundance of the *AOB* gene was higher than the abundance of the *AOA* gene in most of the sampling sites.

Copy numbers of the *nirS* gene and *nosZI* genes ranged from 1.89 × 10^7^ to 1.49 × 10^8^ copies g^−1^ of sediment and from 4.84 × 10^7^ to 4.85 × 10^8^ copies g^−1^ of sediment, respectively (Figures 4C,D). The abundance of the *nosZI* gene exceeded the abundance of the *nirS* gene at most sites. There was a decreasing trend in the abundance of the *nirS* gene and *nosZI* gene from KRE to NCA.

### The Community Structure of Denitrifying Microorganisms

**Figure 5:**
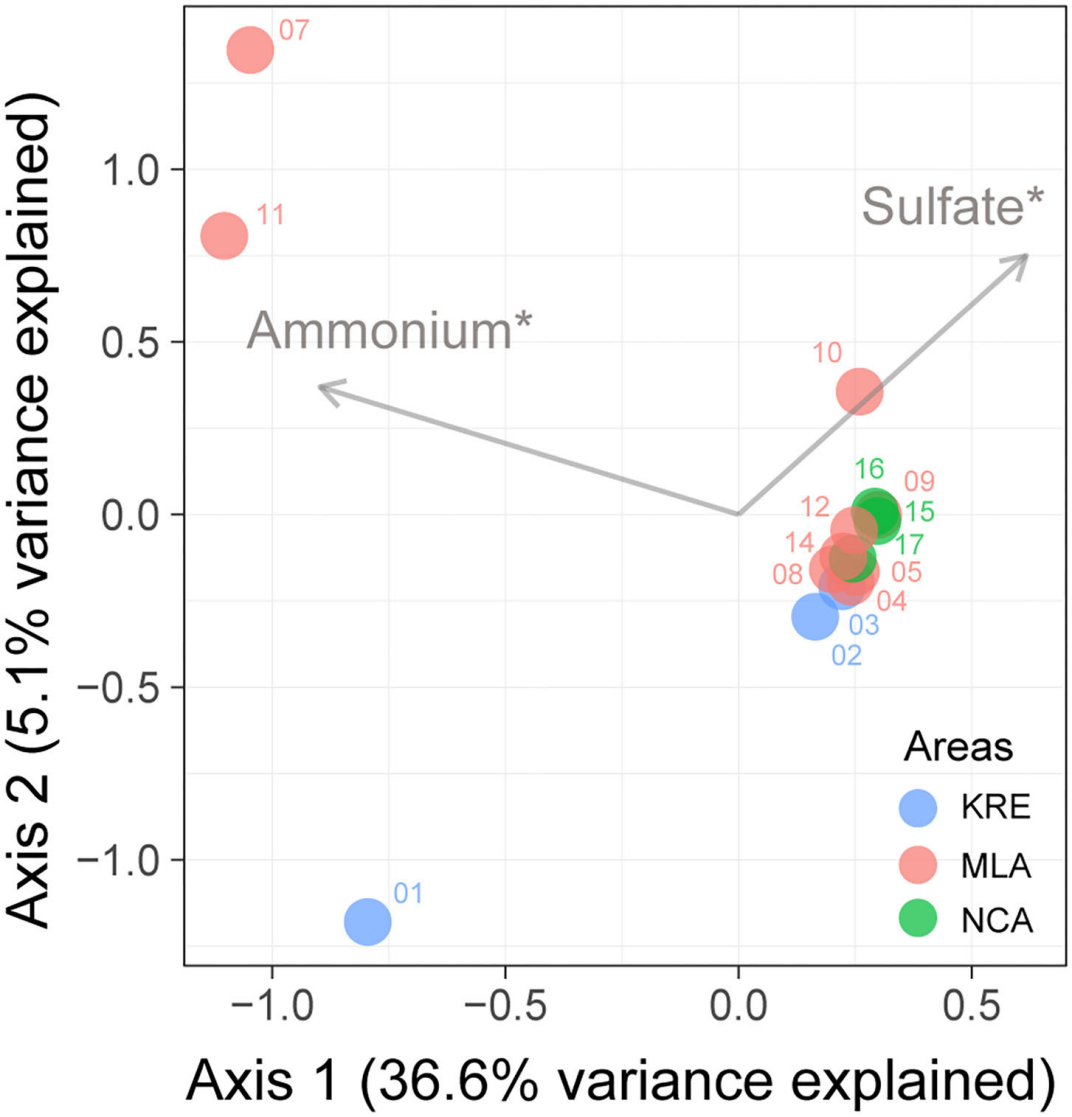
Redundancy analyses (RDA) ordination plots showing the significant environmental factors in structuring variations in the community composition of nitrous oxide reducer in Lake Bosten. Significance levels: *corrected *p*-value < 0.05. KRE, Kaidu River Estuary; MLA, Main Lake Area; NCA, Northwestern Coastal Area.

Alpha-diversity analysis revealed a decreasing trend in the diversity indexes of Richness, Shannon, and Simpson from KRE to NCA (Supplementary Figure S2). Unconstrained principal coordinates analysis (PCoA) with Bray–Curtis distance showed the community variation of nitrous oxide reducers among different sampling sites (Supplementary Figure S3). The results showed that the first two axes explained 87.3% of the microbial community variation. The microbial community of nitrous oxide reducers was significant differences among the three areas in Lake Bosten (*p* < 0.05). The forward selection procedure in RDA revealed that the variation in the microbial community of nitrous oxide reducer was significantly explained by salinity and ammonium, which described 21.3 and 21.5% of the total variation, respectively (Figure 5).

## Discussion

### Nitrate Availability Limited Unamended Denitrification Rates

The main objective of this study was to explore the denitrification potential of Lake Bosten and its response to increasing inputs of nutrients and changes in salinity. Amended denitrification rates increased by one order of magnitude compared to unamended rates in response to additional nitrate at most of the sites, indicating the high potential of denitrification in Lake Bosten. The availability of nitrate is considered one of the most important limiting factors for denitrification in lakes and other aquatic ecosystems (Pina-Ochoa and Alvarez-Cobelas, 2006; Seitzinger et al., 2006). In Lake Bosten, nitrate concentrations in the water positively correlated with unamended denitrification rates (Table 1), indicating that low nitrate concentrations may limit unamended denitrification rates. Nitrate can be supplied as an external input or by *in situ* nitrification. The coupled nitrification-denitrification process usually dominates N removal in ecosystems of nitrate limitation (Koop-Jakobsen and Giblin, 2010; Xia et al., 2017). In Lake Bosten, the abundance of either the *AOB* or *AOA* gene was low compared to the equivalent values in most freshwater lakes by at least one order of magnitude (Wu et al., 2010; Hou et al., 2013; Zhao et al., 2013a; Bollmann et al., 2014; Liu et al., 2014). Meanwhile, the concentrations of ammonium were significantly higher than those of nitrate, perhaps indicating that nitrification was inhibited. Therefore, both the low nitrate concentrations and abundances of nitrifiers may limit denitrification rates in Lake Bosten.

**Table 1:** Pearson's correlation analysis between denitrification rates (amended and unamended) and environmental variables (*n* = 17) and between functional gene abundance (nirS, *nosZI, AOB*, and *AOA*) and environmental variables (*n* = 15).

	**Denitrification rates**		**Functional gene abundance**			
	**Amended**	**Unamended**	***nirS***	***nosZI***	***AOB***	***AOA***
Nitrate	0.41	0.51^*^	0.37	0.66^**^	0.02	−0.01
Ammonium	0.10	0.10	0.13	0.24	−0.07	0.20
TN	0.28	0.51^*^	0.39	0.49	0.08	−0.09
Sulfate	−0.59^*^	−0.44	−0.69^**^	−0.81^**^	−0.11	−0.17
DOC	−0.14	−0.21	−0.54^*^	−0.36	0.06	−0.07
STOC	−0.62^**^	−0.26	−0.40	−0.53^*^	−0.31	−0.22
STN	−0.61^**^	−0.25	−0.34	−0.47	−0.31	−0.13
AVS	−0.53^*^	0.04	−0.45	−0.24	−0.36	−0.26
WT	0.25	0.03	0.20	−0.03	0.15	0.41
pH	0.12	−0.22	−0.12	−0.25	0.51^*^	−0.01
DO	−0.56^*^	−0.13	−0.57^*^	−0.32	−0.24	−0.39
Salinity	−0.50^*^	−0.48	−0.49	−0.62^**^	0.01	−0.08

*The coefficients (r) are shown. Significance levels:*

* *p-value < 0.05,*

** *p-value < 0.01. Environmental variables include nitrate, ammonium, total nitrogen (TN), sulfate, dissolved organic carbon (DOC), sediment total organic carbon (STOC), sediment total nitrogen (STN), acid volatile sulfides (AVS), water temperature (WT), pH, dissolved oxygen (DO) and salinity*.

### The Influence of Changes in Salinity on Denitrification

In Lake Bosten, there is heterogeneity in denitrification potential. The sediments in KRE had the highest amended denitrification rates. Nitrate loads mainly derive from the transportation of the Kaidu River in Lake Bosten (Yu et al., 2015; Zhou et al., 2017), which exhibited an obvious decline in nitrate from the estuary to the east section (Figure 2). This phenomenon indirectly reflects a high potential of N removal in KRE. In contrast, the denitrification potential in NCA was low, suggesting that other environmental factors constrained denitrification except for nitrate availability.

Amended denitrification rates were negatively correlated with salinity and sulfate concentrations in Lake Bosten (Table 1), perhaps indicating that salinity is a key regulating factor of denitrification. Climate change is affecting the hydrological cycle of Lake Bosten and consequent effects on concentration or dilution will alter lake salinity (Rusuli et al., 2015; Liu and Bao, 2020). In addition, there is a continuous input of salinity from salt leaching associated with the agricultural irrigation of the northwestern basin (Tang et al., 2012; Guo et al., 2015b). In the past 60 years, Lake Bosten has evolved from freshwater to an oligosaline lake. Salinity has become a dominant factor shaping the bacterial community in the water and sediment of Lake Bosten (Tang et al., 2012; Dai et al., 2013).

Salinity can affect denitrification in many respects. Considerable studies have reported that salinity directly inhibits the metabolic activity of denitrification, through osmotic stress and sulfide toxicity (Ruiz-Romero et al., 2009; Ardon et al., 2013; Zhao et al., 2013b; Neubauer et al., 2019). Sulfate is the main component of salt anions in Lake Bosten, which is consistent with the changes in salinity (*r* = 0.83, *p* < 0.01). High sulfate can enhance the accumulation of toxic sulfide in sediments (Aelion and Warttinger, 2010; Zhu et al., 2018; Murphy et al., 2020) and then inhibit denitrification rates. Furthermore, the influence of salinity on denitrification depends on the long-term salinity adaptation of denitrifying microorganisms (Franklin et al., 2017; Wang et al., 2018a; Murphy et al., 2020). Many studies have shown that salinity is the most important factor in structuring communities of denitrifying microorganisms, such as *nosZ*-denitrifiers (Cai et al., 2019; Wang et al., 2019; Han et al., 2021), and that salinity can affect denitrification rates by altering the abundance and diversity of denitrifying microorganism. In Lake Bosten, there was a significant correlation between the abundance of the *nirS* and *nosZI* genes (*r* = 0.79, *p* < 0.01). Salinity or sulfate was negatively correlated with the abundance of *nirS* and *nosZI*, respectively, and the latter was the stronger. The nitrous oxide reducers are more sensitive to osmotic stress and ionic toxicity than the nitrite reducers (Laverman et al., 2007; Zhou et al., 2016). In addition, the species diversity of nitrous oxide reducers significantly decreased with the increase of salinity or sulfate in Lake Bosten (*p* < 0.01). Elevated salinity appears to decrease the abundance and diversity of nitrous oxide reducers, resulting in incomplete denitrification.

Salinity also indirectly affects denitrification by inhibiting nitrification rates or altering the physicochemical environment (Herbert et al., 2015; Zhou et al., 2016; Franklin et al., 2017). Previous research also suggests that toxic sulfide can inhibit microbial activity related to nitrification (Herbert et al., 2015; Wang et al., 2020), while some investigations conducted in other habitats showed moderate salinity in favor of nitrification (Zhou et al., 2016; Wang et al., 2018a). In this study, the abundances of *AOB* and *AOA* genes were not correlated with salinity, sulfate, and AVS, indicating that the differences in gene abundances were not attributed to the changes in salinity. Further research is needed to reveal the underlying mechanisms of low gene abundance mediated nitrification in Lake Bosten. In addition, amended denitrification rates were negatively correlated with STOC and DO in Lake Bosten. Elevated salinity might constrain the activities of microbes and reduce the deposition of organic carbon and the consumption of DO, thus inhibiting the anaerobic denitrifying process. Neubauer et al. (2019) also reported that denitrification rates were reduced due to the decrease in oxygen demand induced by salinization.

### The Response of Endorheic Lakes to Climate Change in Northwest China

The arid region of Northwest China has been getting warmer and wetter in recent decades because of the enhancement of the westerly circulation (Yang et al., 2021; Yao et al., 2022). Glacial melt leads to increased runoff, which carries a large amount of silt as well as plant and animal residues (Sorg et al., 2012; Guo et al., 2015b). Meanwhile, the decomposition of organic matter and the release of nutrients may be enhanced by the increasing temperature (Wik et al., 2016; Jane et al., 2021). Thus, climate warming may lead to an increase in nutrient input into lakes from the watershed. For example, climate warming and consequent glacial melt led to the increasing inflow of the Kaidu River into Lake Bosten in 2013 (Guo et al., 2015a). Meanwhile, nitrate concentration in the Kaidu River estuary has been fluctuating but increasing in recent years (Supplementary Figure S4). However, a decreasing trend in nitrate concentration was observed in Lake Bosten. This may be because the increase in water level led to the decrease in salinity and nitrate concentrations through dilution. In addition, the estuary area of the Kaidu River has excess capacity to remove additional nitrate, which plays an important role in regulating and alleviating external nitrate loads, especially with the decrease of salinity.

In Northwest China, the influence of climate change on the water quality of endorheic lakes is complicated. On the one hand, the external nutrient inputs may increase, accompanied by an increase in the quantity of runoff and climate warming, but on the other hand, the pollution will be alleviated due to dilution and the decrease of salinity. Although climate change had a positive effect on the water quality of Lake Bosten so far, long-term trajectories of water quality are difficult to predict. Accelerated climate warming will cause glacier shrinkage and decreased snow cover in the future (Sorg et al., 2012; Zhou et al., 2015). This brings large uncertainties into predictions of the changes in runoff, water level, and salinity. Effective management decisions are essential for maintaining the water quantity, salinity, and denitrification capacity of lakes in the face of accelerated climate change and extensive human activities.

## Data Availability Statement

The data presented in the study are deposited in the China National Center for Bioinformation, accession number CRA006560 (https://www.cncb.ac.cn/).

## Author Contributions

XJ: conceptualization, methodology, investigation, writing—original draft, visualization, and funding acquisition. CL and YH: investigation and data curation. KS: formal analysis and project administration. XT: writing—review and editing. GG and BQ: conceptualization, writing—review andediting, and funding acquisition. All authors contributed to the article and approved the submitted version.

## Funding

This study was financially supported by the National Natural Science Foundation of China (grant numbers: 41790423 and U2003205) and National Key R&D Program of China (grant numbers: 2019YFA0607100).

## Conflict of Interest

The authors declare that the research was conducted in the absence of any commercial or financial relationships that could be construed as a potential conflict of interest.

## Publisher's Note

All claims expressed in this article are solely those of the authors and do not necessarily represent those of their affiliated organizations, or those of the publisher, the editors and the reviewers. Any product that may be evaluated in this article, or claim that may be made by its manufacturer, is not guaranteed or endorsed by the publisher.
