# Supplementary material for: Salinity-Linked Denitrification Potential in Endorheic Lake Bosten (China) and Its Sensitivity to Climate Change
Source: Front Microbiol. 2022 Jul 14;13:922546. doi: 10.3389/fmicb.2022.922546 (PMC9329126; doi:10.3389/fmicb.2022.922546)
Supplement: Supplementary file 1 [file Data_Sheet_1.docx]

Supplementary Material

# Supplementary Tables

**Table S1** Coordinates, water temperature (WT), pH and depth of each sampling site in Lake Bosten

|  | Longitude | Latitude | WT (℃) | pH | Depth (m) |
| --- | --- | --- | --- | --- | --- |
| BST1 | 86.742 | 41.889 | 23.5 | 8.99 | 3.1 |
| BST2 | 86.769 | 41.894 | 24.5 | 8.89 | 7.9 |
| BST3 | 86.850 | 41.889 | 24.4 | 9.09 | 7.5 |
| BST4 | 86.983 | 41.914 | 24.6 | 9.44 | 7.7 |
| BST5 | 87.133 | 41.953 | 24.4 | 9.11 | 10.2 |
| BST6 | 87.275 | 41.919 | 24.3 | 8.97 | 15.6 |
| BST7 | 87.237 | 41.959 | 24.9 | 9.14 | 13.0 |
| BST8 | 87.133 | 42.000 | 24.0 | 9.16 | 14.2 |
| BST9 | 87.133 | 42.047 | 23.6 | 9.06 | 6.5 |
| BST10 | 87.017 | 42.056 | 23.9 | 8.89 | 9.1 |
| BST11 | 86.963 | 42.000 | 24.5 | 9.21 | 11.0 |
| BST12 | 86.906 | 42.006 | 23.7 | 9.27 | 10.1 |
| BST13 | 86.772 | 41.950 | 24.6 | 9.07 | 4.7 |
| BST14 | 86.844 | 41.983 | 24.5 | 8.77 | 4.7 |
| BST15 | 86.872 | 42.042 | 24.4 | 8.97 | 6.4 |
| BST16 | 86.900 | 42.072 | 23.7 | 9.46 | 7.0 |
| BST17 | 86.842 | 42.100 | 23.4 | 9.32 | 3.9 |

**Table S2** The quality of DNA extracted in each sampling site.

| Sampling sites | Nucleic acid (ng uL^-1^) | A260/A280 |
| --- | --- | --- |
| BST01 | 123.685 | 1.850 |
| BST02 | 108.236 | 1.841 |
| BST03 | 193.107 | 1.825 |
| BST04 | 183.393 | 1.847 |
| BST05 | 108.950 | 1.838 |
| BST06 | 0.258 | 0.257 |
| BST07 | 190.701 | 1.868 |
| BST08 | 99.888 | 1.797 |
| BST09 | 101.631 | 1.842 |
| BST10 | 98.359 | 1.816 |
| BST11 | 171.362 | 1.859 |
| BST12 | 152.567 | 1.858 |
| BST13 | -0.065 | -0.093 |
| BST14 | 152.849 | 1.886 |
| BST15 | 141.925 | 1.877 |
| BST16 | 185.407 | 1.900 |
| BST17 | 146.699 | 1.854 |

**Table S3** Primers and qPCR protocols used in the present study.

| Target gene | Primer name | Sequence (5’-3’) | PCR conditions | Reference |
| --- | --- | --- | --- | --- |
| *AOBamoA* | amoA-1F | GGGGTTTCTACTGGTGGT | 95 °C for 2 min, 35 × [95 °C for 15 s, 57 °C for 20 s, 72 °C for 30 s], 72 °C for 2 min | (Rotthauwe et al. 1997) |
|  | amoA-2R | CCCCTCKGSAAAGCCTTCTTC |  |  |
| *AOAamoA* | Arch-amoAF | STAATGGTCTGGCTTAGACG | 95 °C for 2 min, 35 × [95 °C for 15 s, 55 °C for 20 s, 72 °C for 30 s], 72 °C for 2 min | (Francis et al. 2005) |
|  | Arch-amoAR | GCGGCCATCCATCTGTATGT |  |  |
| *nirS* | nirSCd3aF | GTSAACGTSAAGGARACSGG | 95 °C for 2 min, 40 × [95 °C for 15 s, 57 °C for 20 s, 72 °C for 30 s], 72 °C for 2 min | (Throback et al. 2004) |
|  | nirSR3cd | GASTTCGGRTGSGTCTTGA |  |  |
| *nosZ**I* | nosZ-F | CG(C/T)TGTTC(A/C)TCGACAGCCAG | 95 °C for 2 min, 40 × [95 °C for 15 s, 53 °C for 20 s, 56 °C for 30 s], 72 °C for 2 min | (Throback et al. 2004) |
|  | nosZ1622R | CGC(G/A)A(C/G)GGCAA(G/C)AAGGT(G/C)CG |  |  |

**Table S4** qPCR calibration curve parameters in the present study.

| Target gene | Product length (bp) | Slope | y-Intercept | Amplification  Efficiency (%) | R^2^ |
| --- | --- | --- | --- | --- | --- |
| *AOBamoA* | 491 | -3.64 | 35.19 | 88.2 | 0.9916 |
| *AOAamoA* | 635 | -3.44 | 39.34 | 95.4 | 0.9881 |
| *nrfA* | 67 | -3.13 | 38.98 | 108.6 | 0.9901 |
| *nirS* | 425 | -3.54 | 36.26 | 91.8 | 0.9902 |
| *nosZI* | 450 | -3.83 | 37.60 | 82.4 | 0.9963 |

# Supplementary Figures


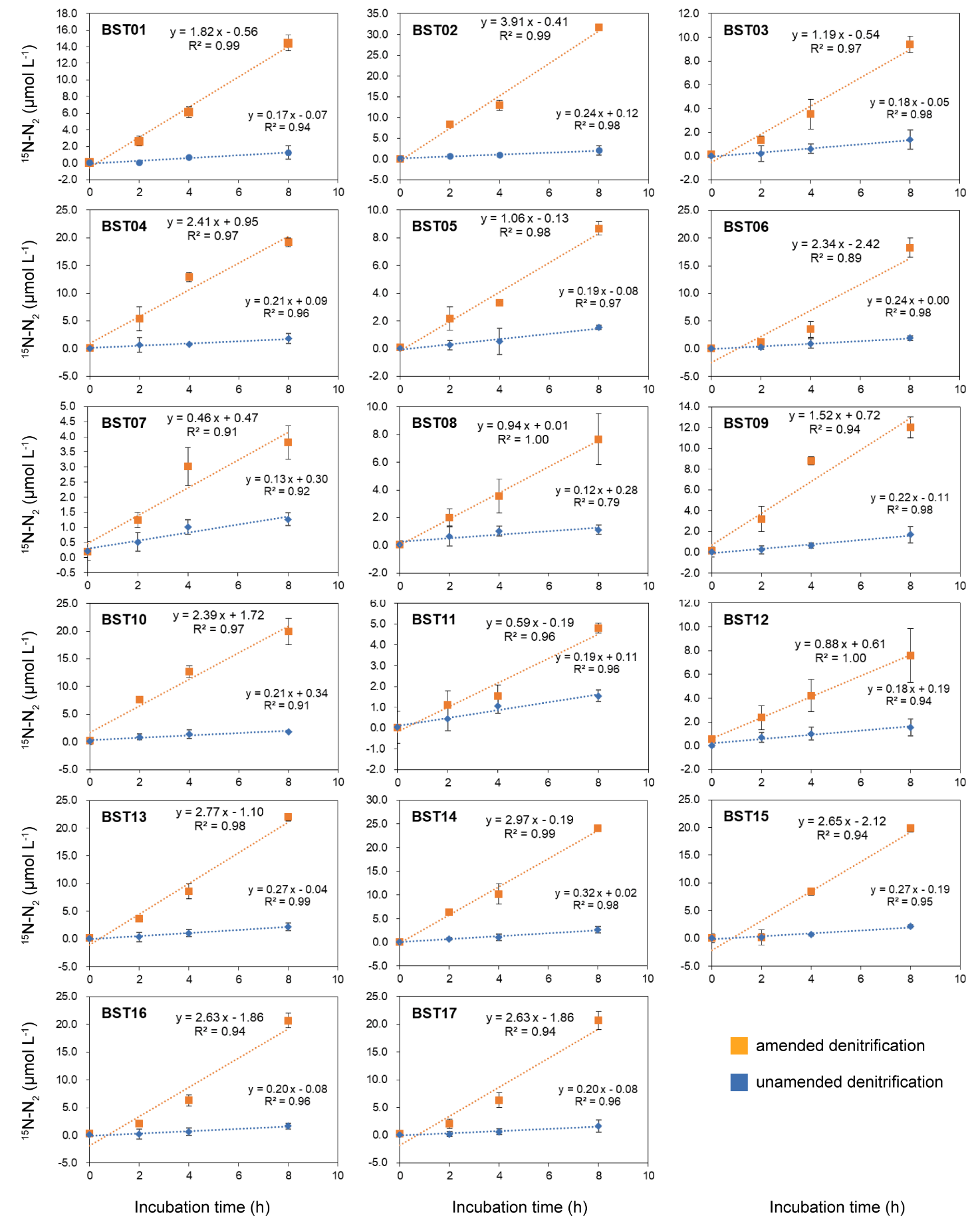


**Figure S1.** The concentrations of ^15^N-N_2_ produced by amended and unamended denitrification during incubation periods.


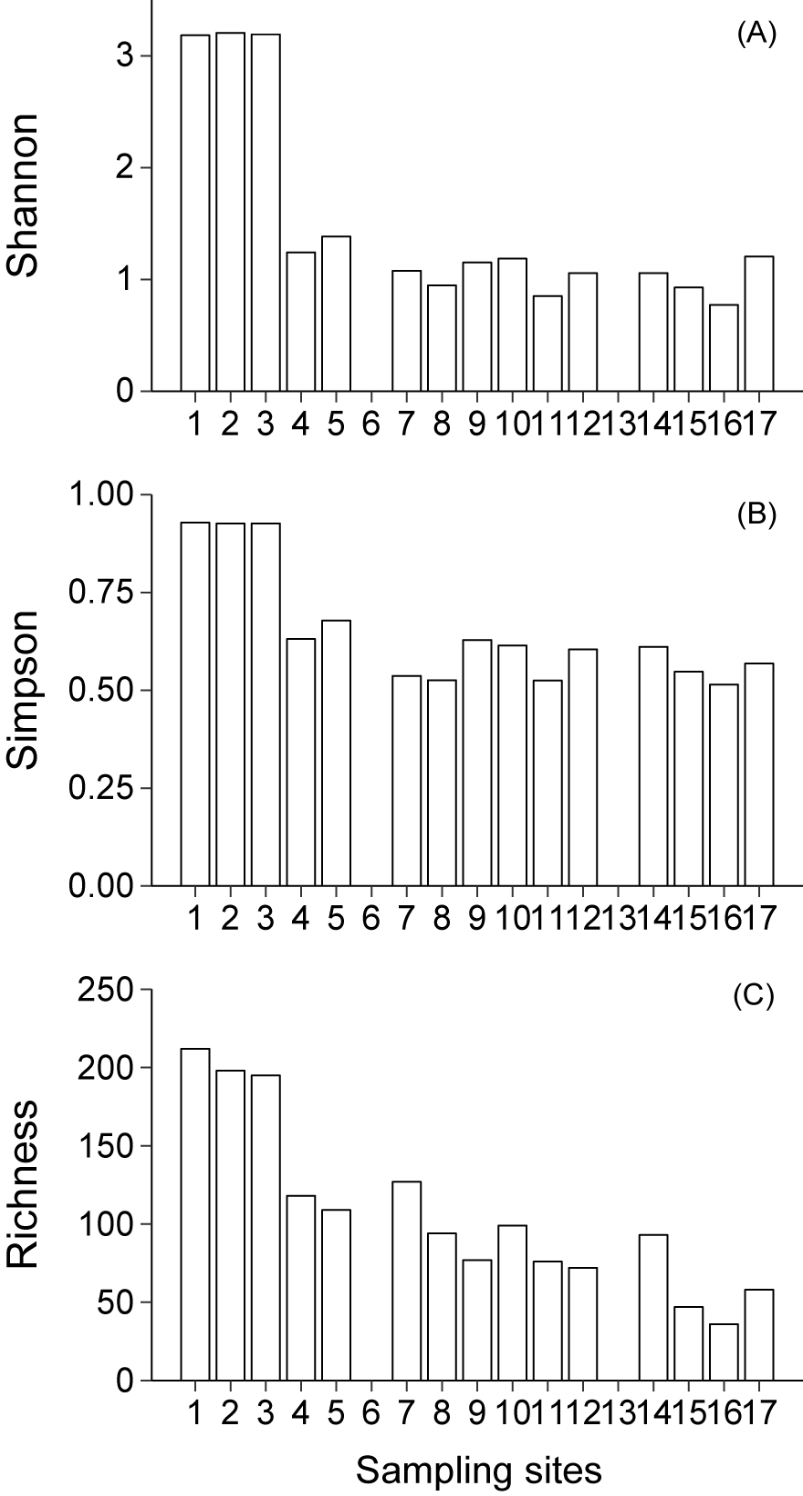


**Figure S2.** Spatial distribution of α-diversity indexes (Richness, Shannon and Simpson) of nitrous oxide reducers in Lake Bosten.


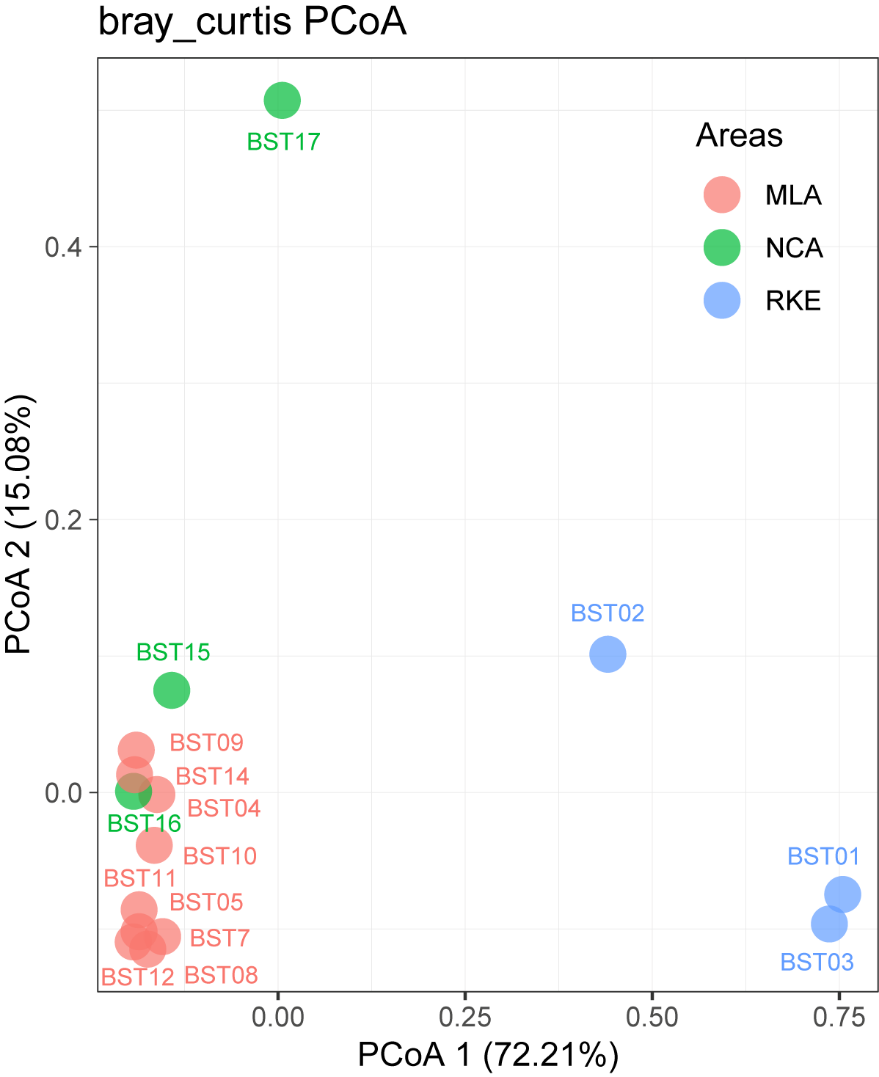


**Figure S3.** Unconstrained PCoA with Bray–Curtis distance showing that the community of nitrous oxide reducers among the three sampling areas in Lake Bosten using adonis analysis.


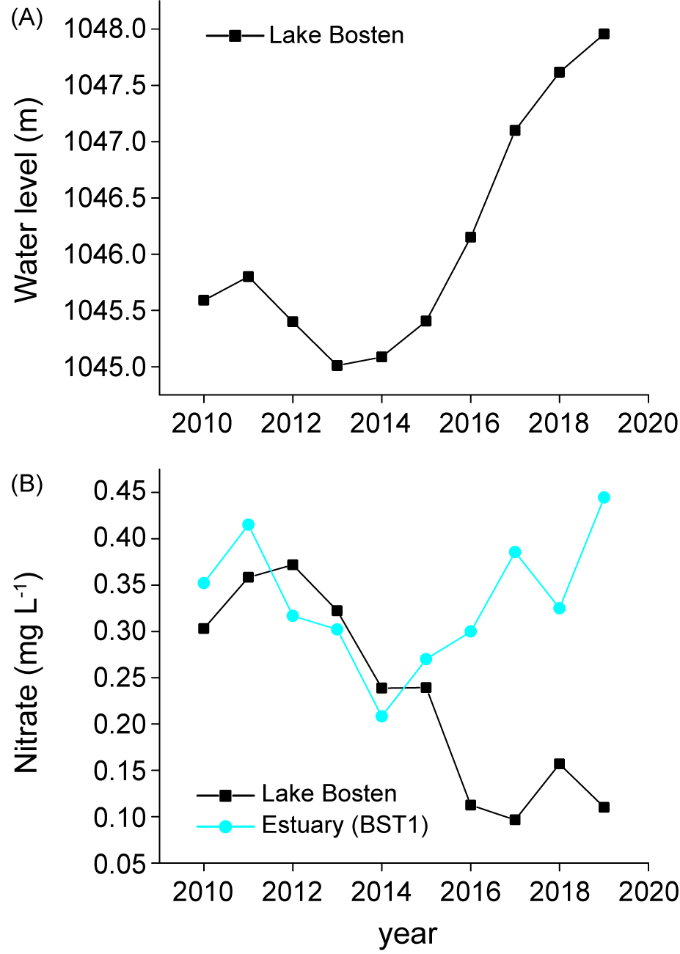


**Figure S4.** (A) The interannual variation of annual mean water level in Lake Bosten. (B) The interannual variation of annual mean nitrate concentration in Lake Bosten and BST1 of Kaidu River Estuary (KRE). Data were derived from the long-term monitoring of the Institute of Lake Bosten.
